# Semaphorin 4D induces vaginal epithelial cell apoptosis to control mouse postnatal vaginal tissue remodeling

**DOI:** 10.3892/mmr.2014.2773

**Published:** 2014-10-27

**Authors:** TAKUJI ITO, TAO BAI, TETSUJI TANAKA, KENJI YOSHIDA, TAKASHI UEYAMA, MASAYASU MIYAJIMA, TAKAYUKI NEGISHI, TAKAHIKO KAWASAKI, HYOTA TAKAMATSU, HITOSHI KIKUTANI, ATSUSHI KUMANOGOH, KAZUNORI YUKAWA

**Affiliations:** 1 Department of Physiology, Faculty of Pharmacy, Meijo University, Tempaku, Nagoya 468-8503, Japan; 2 Department of Obstetrics and Gynecology, Wakayama Medical University, Wakayama 641-8509, Japan; 3 Department of Anatomy, Wakayama Medical University, Wakayama 641-8509, Japan; 4 Institute for Animal Experimentation, Wakayama Medical University, Wakayama 641-8509, Japan; 5 Division of Brain Function, National Institute of Genetics, Graduate University for Advanced Studies (Sokendai), Mishima 411-8540, Japan; 6 Department of Immunopathology, Research Institute for Microbial Diseases, Osaka University, Suita, Osaka 565-0871, Japan; 7 Department of Molecular Immunology, Research Institute for Microbial Diseases, Osaka University, Suita, Osaka 565-0871, Japan

**Keywords:** semaphorin, axon guidance, growth cone collapse, apoptosis

## Abstract

The opening of the mouse vaginal cavity to the skin is a postnatal tissue remodeling process that occurs at approximately five weeks of age for the completion of female genital tract maturation at puberty. The tissue remodeling process is primarily composed of a hormonally triggered apoptotic process predominantly occurring in the epithelium of the distal section of the vaginal cavity. However, the detailed mechanism underlying the apoptotic induction remains to be elucidated. In the present study, it was observed that the majority of BALB/c mice lacking the class 4 semaphorin, semaphorin 4D (Sema4D), developed imperforate vagina and hydrometrocolpos resulting in a perpetually unopened vaginal cavity regardless of a normal estrogen level comparable with that in wild-type (WT) mice. Administration of β-estradiol to infant Sema4D-deficient (Sema4D^−/−^) mice did not induce precocious vaginal opening, which was observed in WT mice subjected to the same β-estradiol administration, excluding the possibility that the closed vaginal phenotype was due to insufficient estrogen secretion at the time of vaginal opening. In order to assess the role of Sema4D in the postnatal vaginal tissue remodeling process, the expression of Sema4D and its receptor, plexin-B1, was examined as well as the level of apoptosis in the vaginal epithelia of five-week-old WT and Sema4D^−/−^ mice. Immunohistochemical analyses confirmed the localization of Sema4D and plexin-B1 in the mouse vaginal epithelia. Terminal deoxynucleotidyl transferase dUTP nick end labeling assay and immunohistochemistry detecting activated caspase-3 revealed significantly fewer apoptotic cells *in situ* in the vaginal mucosa of five-week-old Sema4D^−/−^ mice compared with WT mice. The addition of recombinant Sema4D to Sema4D^−/−^ vaginal epithelial cells in culture significantly enhanced apoptosis of the vaginal epithelial cells, demonstrating the apoptosis-inducing activity of Sema4D. The experimental reduction of plexin-B1 expression in vaginal epithelial cells demonstrated the integral role of plexin-B1 in Sema4D-induced apoptotic cell death. These results suggest a non-redundant role of Sema4D in the postnatal tissue remodeling process in five-week-old BALB/c mice, which involves the induction of vaginal epithelial cell apoptosis through Sema4D binding to plexin-B1.

## Introduction

The development of the mouse female genitalia is primarily completed through a postnatal tissue remodeling process in which the blind-ending vaginal cavity opens to the skin in accordance with the rapid increase of sex hormones in the five-week-old female mouse internal environment (1). The postnatal tissue remodeling process is largely dependent on massive mucosal apoptosis in the distal section of the mouse vaginal cavity near the skin, and can only be observed during the brief period of vaginal opening (1). Transgenic mice lines expressing the human anti-apoptotic protein, B-cell lymphoma 2 (Bcl-2), in their vaginal mucosa developed a closed vaginal phenotype due to the failure of the vaginal epithelium to execute apoptosis, indicating that vaginal mucosal apoptosis is crucial for postnatal vaginal opening at approximately five weeks of age (1). Thereafter, several knockout mice studies revealed the involvement of proapoptotic Bcl-2 family proteins (2,3) and other molecules in postnatal vaginal tissue remodeling (4,5). However, the exact mechanism by which extensive apoptosis is induced in the vaginal epithelium in response to a rapid increase in estrogen in the mouse internal environment at puberty remains to be elucidated (1,6). In particular, little information is available as to whether an apoptosis-inducing ligand is important in the postnatal vaginal tissue remodeling process (1,6). The present study found that imperforate vagina and hydrometrocolpos occurred with a high incidence in mice lacking semaphorin 4D (Sema4D), a member of the semaphorin family known to guide neuronal axon extension during nervous system development (7,8).

Semaphorins can be divided into eight classes, composing a family of soluble and transmembrane glycoproteins with a phylogenetically conserved domain structure, and were originally identified as chemorepellents for axon guidance in the developing nervous system (9). Sema4D, also termed CD100, is a class 4 transmembrane-type semaphorin that induces repulsive cytoskeletal changes in growth cone collapse in hippocampal neurons and retinal ganglial cells in culture (10). To trigger these alterations, Sema4D binds to the transmembrane receptor plexin-B1, a member of the plexin family (10). Sema4D and plexin-B1 have a conserved sema domain of ~400 amino acids characterized by a seven-bladed β-propeller fold in their respective extracellular domains (9,11). Sema4D as a ligand and plexin-B1 as a receptor mutually interact via their respective sema domains as demonstrated by a previous crystallographic study (12). Sema4D binding to plexin-B1 induces clustering of plexin-B1 receptors and then accelerates the GTPase-activating protein (GAP) activities granted by two GAP domains in the intracellular region of plexin-B1 (13). The activities of plexin-B1 GAP downregulate the activities of Ras family members, including R-Ras and M-Ras, which in cultured neurons lowers an integrin-mediated cell attachment to the extracellular matrix and thus induces remodeling of growth cone or dendrite morphology (13,14). Sema4D binding to plexin-B1 also stimulates the guanine nucleotide exchange factor (GEF) activities of PDZ-RhoGEF and leukemia-associated RhoGEF bound to the plexin-B1 C terminal PDZ-binding motif, which facilitates conversion of RhoA from the GDP-bound form to the GTP-bound from (10). The increase in GTP-bound RhoA augments actomyosin contractility through Rho kinase activation and myosin light chain phosphorylation, which also facilitates Sema4D-induced growth cone collapse in cultured hippocampal neurons (8,10).

The high incidence of the closed vaginal phenotype in Sema4D-deficient (Sema4D^−/−^) mice implies a degree of impairment in vaginal mucosal apoptosis at the vaginal opening. Previous studies have demonstrated that Sema4D is involved in the apoptotic induction of neural precursor cells and oligodendrocytes in a cultured model (15,16). However, it remains to be elucidated whether Sema4D is crucially implicated in the apoptosis of the postnatal vaginal tissue remodeling process. Thus, the present study aimed to examine the possible involvement of the semaphorin protein Sema4D in vaginal epithelial apoptosis in the postnatal tissue remodeling process of the female mouse.

## Materials and methods

### Generation of Sema4D^−/−^ mice

Sema4D^−/−^ mice were produced by gene targeting (17). In brief, the procedure for generating the mice was performed as follows. A gene-targeting vector was designed to replace the 1.6-kb genomic region, which contained the putative first exon covering the initiation codon, with a neomycin-resistance gene. The gene-targeting vector was transfected into E14.1 embryonic stem (ES) cells by electroporation. Determination of the homologous recombinants was confirmed by polymerase chain reaction (PCR) and Southern blotting of G418- and ganciclovir-resistant clones. Mutant ES cells with the homologous recombination were introduced into mouse blastocysts and transferred into pseudopregnant mice to generate chimeras. F1 heterozygous knockout mice were generated by breeding the chimeras with BALB/c mice, and were then backcrossed with BALB/c mice for 10 generations. Pairs of resultant heterozygous mice were bred to gain homozygous knockout mice and their wild-type (WT) littermates as controls. The mice were housed in the Wakayama Medical University animal facilities and the animal center at the Faculty of Pharmacy of Meijo University (Tempaku, Nagoya, Japan). All researchers and experimenters conducted the care and sacrifice of mice as well as other experimental protocols in accordance with the guidelines promulgated by the Physiological Society of Japan as well as the guidelines on animal experimentation of Wakayama Medical University and Meijo University. The Animal Ethics Review Committees of these institutions approved the experimental protocol.

### Genotype analysis

The genotypes of the mice were confirmed by PCR with mouse tail DNA as the template and a Sema4D gene specific primer set as previously reported (17).

### Serum estradiol measurement

The estradiol levels in the serum of WT and Sema4D^−/−^ mice were measured using an enzyme immunoassay kit (ERK R7005; Endocrine Technologies, Inc., San Francisco, CA, USA) according to the manufacturer’s instructions.

### Estradiol administration to induce precocious vaginal opening

17β-estradiol (Sigma-Aldrich, St. Louis, MO, USA) was dissolved in ethanol. The ethanolic solution of 17β-estradiol diluted in corn oil (0.1 μg/kg body weight/day) was subcutaneously injected into 12-day-old female WT and Sema4D^−/−^ mice, and the administrations were repeated daily for 5 days. Induction of precocious vaginal opening was examined by visual inspection of 17-day-old estradiol-treated mice.

### Immunohistochemistry and terminal deoxynucleotidyl transferase dUTP nick end labeling (TUNEL) assay

Mice under anesthesia with pentobarbital sodium (0.648 mg/10 g body weight via intraperitoneal injection; Kyoritsuseiyaku Co., Tokyo, Japan) were subjected to transcardiac perfusion of 4% paraformaldehyde. The vaginas were excised from the mice and fixed overnight in 4% paraformaldehyde solution. The vaginas were embedded longitudinally in paraffin and cut into 4-μm serial sections. The sections were immunolabeled with anti-mouse Sema4D (cat.no D142-3; Medical and Biological Laboratories Co., Ltd., Nagoya, Japan), anti-plexin-B1 (cat.no. sc-25642; Santa Cruz Biotechnology, Inc., Santa Cruz, CA, USA) and anti-cleaved caspase-3 antibody (cat.no. #9664; Cell Signaling Technology, Beverly, MA, USA). TUNEL assay was performed, as described previously (18), using a Dead End™ Fluorometric TUNEL system (Promega, Madison, WI, USA) and an ApoTag Peroxidase In Situ Apoptosis Detection kit (Chemicon International, Inc., Temecula, CA, USA) according to the manufacturer’s instructions.

### Western blot analysis

For Western blot analysis, tissue extracts were prepared by homogenizing mouse vaginal tissue in T-PER Tissue Protein Extraction Reagent (Thermo Scientific Inc., Waltham, MA, USA) containing a protease inhibitor (α-complete; Roche Applied Science, Penzberg, Germany) and a phosphatase inhibitor (PhosStop; Roche Applied Science). Protein quantification of the tissue extract was performed using the Bio-Rad Protein Assay (Bio-Rad, Hercules, CA, USA). Each sample (15 μg) was prepared in a final solution of 60 mM Tris-HCl (pH 6.8), 2% sodium dodecyl sulfate (SDS), 10% glycerol, 0.1% bromophenol blue and 5% β-mercaptoethanol. The sample solution was heated at 100°C for 5 min, electrophoresed through a 10% SDS-polyacrylamide gel and transferred onto polyvinylidene fluoride membranes (Amersham Pharmacia Biotech, Buckinghamshire, UK). Sema4D, plexin-B1 and cleaved caspase-3 were detected with their respective antibodies using an enhanced chemiluminescence or enhanced chemiluminescence-plus western blot detection system in accordance with the manufacturer’s instructions (Amersham Pharmacia Biotech). The antibodies used were anti-CD100/Sema4D (cat.no. 610670; BD Transduction Laboratories, Franklin Lakes, NJ, USA), anti-plexin-B1 (cat.no. sc-28372; Santa Cruz Biotechnology, Inc.) and anti-cleaved caspase-3 (cat.no. #9664 Cell Signaling Technology).

### Mouse vaginal epithelial cell culture

Primary vaginal epithelial cell cultures derived from Sema4D^−/−^ mice were grown according to the procedure developed previously (19). Four-week-old mouse vaginal tissue was incubated with 1% collagenase solution (Wako Pure Chemical Industries, Ltd., Osaka, Japan) at 37°C for 60 min to prepare an epithelial sheet that was later cut into small pieces and dispersed into individual cells by trypsin treatment. The dispersed vaginal epithelial cells were grown on a 10-cm Primaria™-treated tissue culture dish (BD, Tokyo, Japan) in Dulbecco’s modified Eagle’s medium supplemented with 10% heat-inactivated fetal calf serum and maintained at 37°C with a 5% CO_2_, 95% air atmosphere. Following 5 days of culture, the cells were collected and seeded on poly-L-lysine/laminin-coated coverslips with a density of 2×10^4^ cells per 1 ml of culture medium. Recombinant soluble mouse Sema4D fused to IgG1-Fc (20) was added to the culture at a concentration of 2 μg/ml and after 36 h, the vaginal cell cultures were fixed with 4% paraformaldehyde solution. The fixed cells were subjected to TUNEL assay and anti-cleaved caspase-3 immunocytochemistry to examine apoptosis. To reduce the expression of the plexin-B1 receptor on cultured vaginal epithelia cells via gene knockdown, the vaginal epithelial cell cultures were transduced at a multiplicity of infection of 2.5 with Mission Sigma Lentiviral particles expressing short hairpin RNA (shRNA) directed against mouse plexin-B1 mRNA (Clone ID: NM_172775.1-6159s1c1; Sigma-Aldrich) and examined for Sema4D-induced apoptosis. The vaginal epithelial cell culture transduced with non-target shRNA (shRNA-NT) lentiviral particles (SHC002V; Sigma-Aldrich) was used as a control. The knockdown of plexin-B1 mRNA was confirmed by quantitative PCR using QuantiTect Primer assays according to the manufacturer’s instructions (Mm_Plxnb1_1_SG QT00126483 and Mm_B2m_2_SG QT01149547; Qiagen, Tokyo, Japan).

### Statistical analysis

All data values are presented as the mean ± standard error of the mean. Comparisons between WT and Sema4D^−/−^ mice were performed using Student’s t-test or one-way analysis of variance followed by post-hoc analysis. P<0.05 was considered to indicate a statistically significant difference.

## Results

### Sema4D^−/−^ mice exhibit a closed vaginal phenotype despite normal levels of estrogen

**Figure 1:**
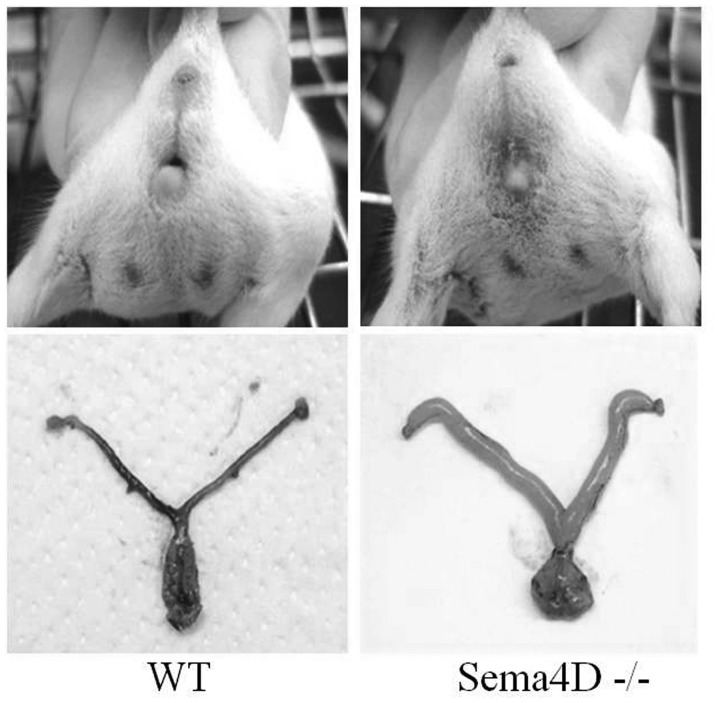
Sema4D deficiency induces a closed vagina phenotype in BALB/c mice. During inspection, no vaginal orifice in the skin of the genital area in female Sema4D^−/−^ mice was identified. This obstruction resulted in swelling around the genital region caused by severely distended genital tracts disclosed by the resection of the uterus and vagina. Sema4D, semaphorin 4D; WT, wild-type.

Sema4D^−/−^ mice with the BALB/c genetic background were generated by a homologous recombination method as previously described (17). The majority of the female Sema4D^−/−^ mice showed lower abdominal distention and swelling of the genital area, causing an appearance similar to that of the male scrotum (Fig. 1). During inspection of the genital areas, no vaginal openings to the skin were identified (Fig. 1). Anatomical dissections of these mice revealed hydrometrocolpos, a condition where the absence of a vaginal opening leads to lower abdominal and genital swelling caused by the over-retention of secreted fluid in distended genital tracts (Fig. 1). The incidence of such imperforate vaginas was significantly higher in Sema4D^−/−^ mice compared with WT mice (WT: 0%, n=80; heterozygous: 7.3%, n=288; Sema4D^−/−^: 59.5%, n=279; χ^2^-test, P<0.05).

**Figure 2:**
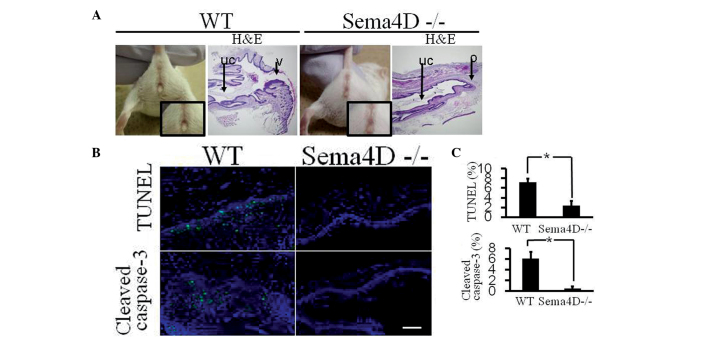
Precocious vaginal opening is not induced by β-estradiol injection in Sema4D^−/−^ mice. (A) Daily subcutaneous injection of β-estradiol (0.1 μg/kg body weight) into WT mice for five consecutive days (between 12 and 16-days-old) induced precocious vaginal opening at 17 days old (vaginal opening: four out of four mice). Identical injection into Sema4D^−/−^ mice did not induce precocious vaginal opening at 17 days old, which was further indicated by histological analysis (vaginal opening: zero out of four mice). (Magnification, ×40). uc, uterogenital canal; o, obstruction; v, external vaginal entrance; H&E, hematoxylin and eosin staining. (B) TUNEL-positive and cleaved caspase-3-positive apoptotic cells (green) were significantly less numerous in the vaginal epithelium of β-estradiol-injected Sema4D^−/−^ mice than in β-estradiol-injected WT mice. Cellular nuclei were visualized with DAPI (blue). (Magnification, ×400). Scale bar=50 μm. (C) Graphs show the rate of TUNEL- or cleaved caspase-3-positive cells, respectively, among nucleated cells in vaginal epithelia. Each column represents the mean ± standard error of the mean (WT, n=4; Sema4D^−/−^, n=4). ^*^P<0.05. Sema4D, semaphorin 4D; WT, wild-type; TUNEL, terminal deoxynucleotidyl transferase dUTP nick end labeling; DAPI, 4′,6-diamidino-2-phenylindole.

The mouse vaginal opening process is modulated by the level of estrogen (1). An enzyme immunoassay was thus conducted to evaluate the serum estrogen level corresponding to the time of vaginal opening of five-week-old WT and Sema4D^−/−^ mice, and no significant difference was identified between the two genotypes (WT: 19.89±6.80 pg/ml, n=5; Sema4D^−/−^: 23.20±3.92 pg/ml, n=5; Student’s t-test, P>0.05). To further exclude the possibility of insufficient estrogen secretion during the critical period of vaginal opening, β-estradiol was injected into infant mice for five consecutive days beginning at 12 days of age to induce precocious vaginal opening in the 17-day-old specimens (1). Administration of β-estradiol to WT mice induced premature vaginal opening in 17-day-old mice and induce apoptosis in vaginal tissue, which was detected by TUNEL assay and activated caspase-3 immunohistochemistry (Fig. 2). By contrast, administration of β-estradiol to Sema4D^−/−^ mice did not induce premature vaginal opening, and the apoptotic level in Sema4D^−/−^ mouse vaginal tissue was significantly lower than that in the vaginal tissue of WT mice treated with β-estradiol (Fig. 2).

### Sema4D and plexin-B1 are localized to mouse vaginal epithelia

**Figure 3:**
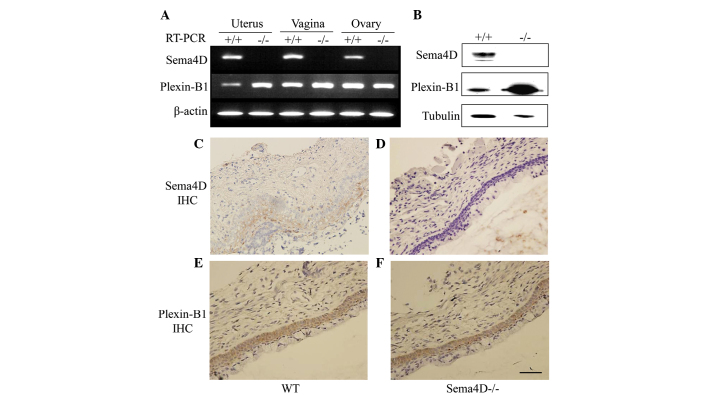
Sema4D and plexin-B1 are expressed in mouse vaginal epithelia. (A) Sema4D mRNA was detected by RT-PCR in the uteri, vaginas and ovaries of WT mice, but not in those of Sema4D^−/−^ mice. Plexin-B1 mRNA was detected by RT-PCR in WT and Sema4D^−/−^ vaginas. +/+, WT mice; −/−, Sema4D^−/−^ mice. (B) Western blotting detected Sema4D in WT vaginas, but not in Sema4D^−/−^ vaginas. Plexin-B1 protein was detected in WT and Sema4D^−/−^ vaginas. +/+, WT mice; −/−, Sema4D^−/−^ mice. (C) Immunohistochemical analyses with anti-Sema4D antibodies detected Sema4D in the suprabasal layer of the vaginal epithelia in WT mice (arrow). (D) IHC did not detect Sema4D in Sema4D^−/−^ vaginas. (E) Plexin-B1 was detected in WT vaginal mucosa by immunohistochemical analysis using plexin-B1-specific antibodies (arrow). (F) Plexin-B1 was also detected in Sema4D^−/−^ vaginal mucosa by IHC (arrow). Scale bar=50 μm. (Magnification of C-F, ×400). Sema4D, semaphorin 4D; WT, wild-type; RT-PCR, reverse transcription-polymerase chain reaction; IHC, immunohistochemistry.

In order to ascertain whether Sema4D mRNA is expressed in the mouse vagina, reverse transcription PCR analyses were performed with RNA from mouse uteri, vaginas and ovaries. Sema4D mRNA was detected in the mouse vagina; however, the transcripts were not amplified in any organs in the Sema4D^−/−^ mice (Fig. 3A). To confirm the existence of Sema4D protein in the mouse vagina, western blotting was performed using protein extracts from WT and Sema4D^−/−^ vaginas (Fig. 3B). The analysis detected Sema4D protein in WT vaginas, but not in Sema4D^−/−^ vaginas (Fig. 3B). Using the same blot, the antibodies against plexin-B1, a Sema4D receptor, revealed the existence of plexin-B1 in WT and Sema4D^−/−^ vaginas (Fig. 3B). To localize the expression of Sema4D and plexin-B1 in the mouse vagina, immunohistochemical analyses were performed on vaginal tissues from WT and Sema4D^−/−^ mice. The antibodies against Sema4D detected Sema4D in the suprabasal layer of the vaginal epithelia in WT mice (Fig. 3C), but not in Sema4D^−/−^ mice (Fig. 3D). However, the plexin-B1 antibodies detected plexin-B1 localization in WT (Fig. 3E) and Sema4D^−/−^ vaginal epithelia (Fig. 3F).

### Fewer apoptotic cells exist in Sema4D^−/−^ vaginal epithelia

**Figure 4:**
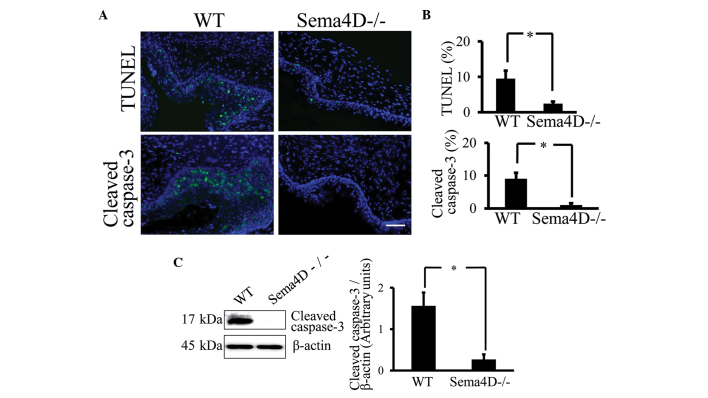
In Sema4D^−/−^ mice, the number of apoptotic cells in the vaginal epithelia is significantly fewer than in WT vaginal epithelia. (A) TUNEL and immunohistochemistry with anti-cleaved caspase-3 antibodies detected apoptotic cells in five-week-old WT mouse vaginal epithelia. Apoptotic cells identified by these methods were hardly detectable in Sema4D^−/−^ vaginal epithelium. Nuclei were visualized with 4′,6-diamidino-2-phenylindole. Scale bar=50 μm. (B) Apoptotic cells detected with TUNEL and cleaved caspase-3 immunohistochemistry were significantly lower in number in Sema4D^−/−^ vaginal mucosa compared with WT vaginal epithelium. WT, wild-type vaginal epithelium; Sema4D−/−, Sema4D^−/−^ vaginal epithelium. ^*^P<0.05. (C) Western blot analysis detected significantly less cleaved caspase-3 in Sema4D^−/−^ vaginal tissue compared with WT vaginal tissue. WT, wild-type vaginal tissue. Sema4D−/−, Sema4D^−/−^ vaginal tissue. ^*^P<0.05. Sema4D, semaphorin 4D; WT, wild-type; TUNEL, terminal deoxynucleotidyl transferase dUTP nick end labeling.

TUNEL assay and cleaved caspase-3 immunohistochemistry was applied to detect apoptotic cells *in situ* and examine apoptosis in the vaginal epithelia of five-week-old WT and Sema4D^−/−^ mice. Several TUNEL-positive and cleaved caspase-3-positive cells were observed in the WT vaginal epithelia (Fig. 4A). By contrast, there were fewer TUNEL-positive and cleaved caspase-3-positive cells in the Sema4D^−/−^ vaginal epithelia (Fig. 4A). Statistical analyses revealed significantly fewer TUNEL-positive and cleaved caspase-3-positive cells in Sema4D^−/−^ vaginal epithelia compared with WT epithelia (Fig. 4B). Western blotting of cleaved caspase-3 confirmed the significantly lower level of apoptosis in the Sema4D^−/−^ vaginal tissues compared with the WT tissues (Fig. 4C).

### Sema4D induces apoptosis in cultured vaginal epithelial cells derived from Sema4D−/− mice

**Figure 5:**
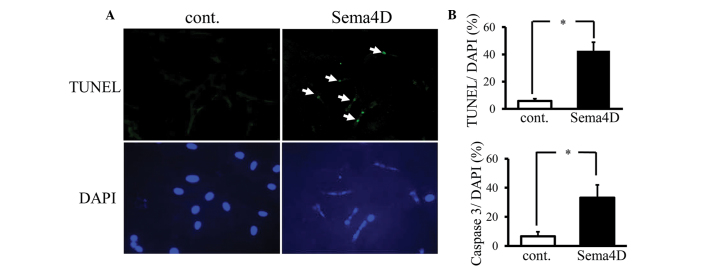
Sema4D induces apoptosis in Sema4D^−/−^ vaginal epithelial cells in culture. (A) Rescue experiments adding recombinant Sema4D to cultured vaginal epithelial cells from Sema4D^−/−^ mice demonstrated that Sema4D induces apoptosis of mouse vaginal epithelial cells as detected by the TUNEL method (arrows). cont., culture without Sema4D; Sema4D, culture with recombinant Sema4D. (Magnification, ×400). (B) Sema4D induced significant apoptosis of vaginal epithelial cells from Sema4D^−/−^ mice, as detected using TUNEL and activated caspase-3 immunohistochemistry. Graph shows the rate of TUNEL-positive or cleaved caspase-3-positive cells among DAPI-positive nucleated cells. ^*^P<0.05. Sema4D, semaphorin 4D; DAPI, 4′,6-diamidino-2-phenylindole; TUNEL, terminal deoxynucleotidyl transferase dUTP nick end labeling.

To examine whether Sema4D induces apoptosis of vaginal epithelial cells, recombinant Sema4D was added to primary vaginal epithelial cells derived from Sema4D^−/−^ mice. After 36 h, Sema4D increased TUNEL-positive cells in culture (Fig. 5A). Quantitative analysis demonstrated that Sema4D induced a significant increase in the percentage of TUNEL-positive vaginal epithelial cells (Fig. 5B). Immunocytochemistry with antibodies against cleaved caspase-3 also demonstrated that vaginal cells with activated caspase-3 were significantly more numerous in Sema4D-treated culture as compared with the untreated culture (Fig. 5B). Thus, Sema4D induced apoptosis of Sema4D^−/−^ vaginal epithelial cells in culture.

### Sema4D-induced apoptosis of vaginal epithelial cells is mediated through plexin-B1

**Figure 6:**
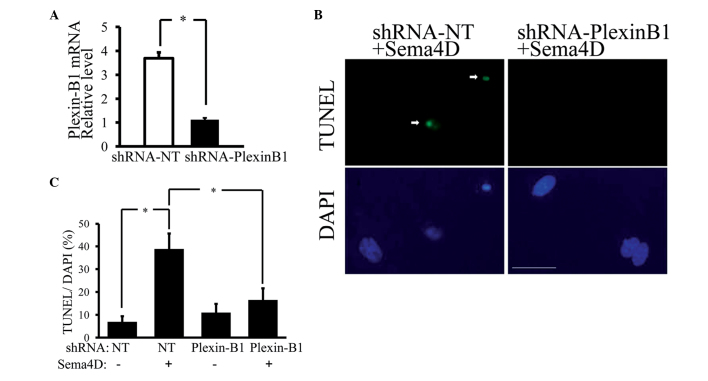
Sema4D-induced vaginal epithelial cell apoptosis is mediated through plexin-B1. (A) Quantitative reverse transcription-polymerase chain reaction confirmed the knock down of plexin-B1 mRNA expression by the lentiviral vector harboring plexin-B1 specific shRNA in cultured vaginal epithelial cells derived from Sema4D^−/−^ mice. ^*^P<0.05, Student’s t-test. (B) Sema4D-induced TUNEL-positive cells were hardly detectable in cultured Sema4D^−/−^ vaginal epithelial cells infected with the lentiviral vector harboring plexin-B1 specific shRNA. shRNA-NT + Sema4D, control shRNA-NT-transduced Sema4D^−/−^ vaginal epithelial cell culture applied with recombinant Sema4D; Plexin-B1 shRNA + Sema4D, Plexin-B1 shRNA-transduced Sema4D^−/−^ epithelial cell culture applied with recombinant Sema4D. Arrow-head indicates TUNEL-positive cells. Scale bar=10 μm. (C) Knockdown of plexin-B1 expression using lentiviral vector harboring plexin-B1 specific shRNA significantly inhibited Sema4D-induced apoptosis in mouse vaginal epithelial cells. The graph shows the ratio of TUNEL-positive cells to DAPI-positive nucleated cells. shRNA-NT, vaginal epithelial cell culture transduced with shRNA-NT; shRNA-Plexin-B1, vaginal epithelial cell culture transduced with plexin-B1 specific shRNA. Sema4D -, without Sema4D. Sema4D +, applied with recombinant Sema4D. ^*^P<0.05. Sema4D, semaphorin 4D; DAPI, 4′,6-diamidino-2-phenylindole; TUNEL, terminal deoxynucleotidyl transferase dUTP nick end labeling; shRNA, short hairpin RNA; shRNA-NT, non-target shRNA.

To investigate whether plexin-B1 is involved in the Sema4D-induced apoptosis of vaginal epithelial cells in culture, lentiviruses with shRNA directed to knockdown plexin-B1 were added to cultured Sema4D^−/−^ vaginal epithelial cells. Two days after lentivirus application, the level of plexin-B1 mRNA was significantly reduced in the vaginal cell culture treated with shRNA against plexin-B1 (Fig. 6A). Sema4D-induced apoptosis was examined on a TUNEL assay 36 h after recombinant Sema4D was added to the culture with plexin-B1 knockdown. As a result, vaginal epithelial cells with plexin-B1 knockdown had significantly fewer TUNEL-positive cells compared with cells harboring control shRNA (Fig. 6B and C). Thus, knockdown of plexin-B1 in vaginal epithelial cells inhibited Sema4D-induced apoptosis, indicating that Sema4D used plexin-B1-mediated cell signaling to execute the apoptosis of vaginal epithelial cells.

## Discussion

The present study analyzed Sema4D^−/−^ BALB/c mice with a closed vaginal phenotype and revealed the importance of Sema4D (a class 4 semaphorin) in the vaginal opening process, a mouse postnatal tissue remodeling phenomenon (1). The present study also revealed the apoptosis-inducing activity of Sema4D in cultured vaginal epithelial cells and the integral role of plexin-B1 for the completion of apoptosis. Thus, to the best of our knowledge, the present study is the first to report the novel physiological role of semaphorin, a known axon guidance molecule, in the mouse postnatal vaginal opening process.

The mouse postnatal vaginal opening process occurring at approximately five weeks old is largely dependent on massive vaginal mucosal apoptosis, which is initiated by rapidly elevated levels of estrogen in the body at the time of postnatal vaginal tissue remodeling (1). Since administration of β-estradiol to infant Sema4D^−/−^ mice did not induce either precocious vaginal opening or vaginal mucosal apoptosis (Fig. 2), Sema4D may be crucially implicated in apoptosis of the vaginal opening process by acting downstream of estrogen during its elevation at mouse puberty. The lower number of apoptotic cells observed in the vaginal epithelia of five-week-old Sema4D^−/−^ mice (Fig. 4) suggests insufficient apoptosis at the time of vaginal opening as the cause of imperforate vagina in Sema4D^−/−^ mice. The high incidence of imperforate vagina and prominent decrease in vaginal epithelial apoptosis during the vaginal opening period in Sema4D^−/−^ mice implies that Sema4D is able to induce apoptosis in vaginal epithelial cells. The present study, by adding recombinant Sema4D to Sema4D^−/−^ vaginal epithelial cells in culture, demonstrated the apoptosis-inducing ability of Sema4D (Fig. 5). Sema3A, a class 3 semaphorin, is known to induce apoptosis in kidney podocytes (21). Sema4D, released from activated T lymphocytes, induces apoptosis in neural progenitor cells and immature oligodendrocytes (15). A previous study suggested that Sema4D is able to regulate the differentiation of oligodendrocytes by facilitating oligodendrocytic apoptosis (16). Furthermore, Sema3A was demonstrated to regulate Fas-mediated apoptosis by promoting migration of the Fas molecule to lipid rafts (22). Thus, during development, semaphorins function not only as axon guidance molecules but also as inducers of apoptosis. The present study revealed the involvement of plexin-B1 in the Sema4D-induced apoptosis of vaginal epithelial cells in culture (Fig. 6). Therefore, Sema4D may promote postnatal vaginal opening by inducing massive vaginal epithelial apoptosis by binding to plexin-B1 receptors on vaginal epithelial cells.

Imperforate vagina has not been observed in Sema4D^−/−^ C57BL/6 mice, although they are present in Sema4D^−/−^ BALB/c mice with a high incidence. In Sema4D^−/−^ C57BL/6 mice, there is a migratory defect of luteinizing-hormone-releasing-hormone neuron precursor cells from the olfactory placode to the hypothalamus during embryonic development (23). Furthermore, there is a significant reduction in the number of secondary ovarian follicles in Sema4D^−/−^ C57BL/6 mice ovaries (24). In the present study, no significant difference was identified in the serum estrogen level between WT and Sema4D^−/−^ BALB/c mice at the time of vaginal opening. The injection of β-estradiol into infant Sema4D^−/−^ mice suggested that the closed vaginal phenotype was not caused by insufficient estrogen secretion in the mutant mice (Fig. 2). Although plexin-B1 was identified as a receptor for the induction of vaginal epithelial cell apoptosis in the present study (Fig. 6), imperforate vagina has not been reported in plexin-B1-deficient C57BL/6 mice (25,26). This may reflect the phenotypic differences dependent on genetic background, where vaginal epithelial cell apoptosis may be more highly dependent on Sema4D/plexin-B1 signaling in BALB/c mice than in other mouse strains. Future studies are required to investigate whether imperforate vagina is also observed in plexin-B1-deficient BALB/c mice.

In conclusion, the results from the present study suggest that the vaginal opening caused through postnatal tissue remodeling in BALB/c mice proceeds as a result of massive epithelial cell apoptosis in the vaginal cavity signaled by Sema4D and plexin-B1 when the mice are five weeks old.
